# Contributions of Mamu-A*01 Status and *TRIM5* Allele Expression, But Not *CCL3L* Copy Number Variation, to the Control of SIVmac251 Replication in Indian-Origin Rhesus Monkeys

**DOI:** 10.1371/journal.pgen.1000997

**Published:** 2010-06-24

**Authors:** So-Yon Lim, Tiffany Chan, Rebecca S. Gelman, James B. Whitney, Kara L. O'Brien, Dan H. Barouch, David B. Goldstein, Barton F. Haynes, Norman L. Letvin

**Affiliations:** 1 Beth Israel Deaconess Medical Center, Harvard Medical School, Boston, Massachusetts, United States of America; 2 Dana Farber Cancer Institute, Harvard Medical School, Boston, Massachusetts, United States of America; 3 Center for Population Genomics and Pharmacogenetics, Duke Institute for Genome Sciences and Policy, Duke University, Durham, North Carolina, United States of America; 4 Duke Human Vaccine Institute, Duke University, Durham, North Carolina, United States of America; Fred Hutchinson Cancer Research Center, United States of America

## Abstract

CCL3 is a ligand for the HIV-1 co-receptor CCR5. There have recently been conflicting reports in the literature concerning whether *CCL3-like gene (CCL3L)* copy number variation (CNV) is associated with resistance to HIV-1 acquisition and with both viral load and disease progression following infection with HIV-1. An association has also been reported between *CCL3L* CNV and clinical sequelae of the simian immunodeficiency virus (SIV) infection *in vivo* in rhesus monkeys. The present study was initiated to explore the possibility of an association of *CCL3L* CNV with the control of virus replication and AIDS progression in a carefully defined cohort of SIVmac251-infected, Indian-origin rhesus monkeys. Although we demonstrated extensive variation in copy number of *CCL3L* in this cohort of monkeys, *CCL3L* CNV was not significantly associated with either peak or set-point plasma SIV RNA levels in these monkeys when MHC class I allele Mamu-A*01 was included in the models or progression to AIDS in these monkeys. With 66 monkeys in the study, there was adequate power for these tests if the correlation of *CCL3L* and either peak or set-point plasma SIV RNA levels was 0.34 or 0.36, respectively. These findings call into question the premise that *CCL3L* CNV is important in HIV/SIV pathogenesis.

## Author Summary

Host genetic factors are important in determining why some individuals maintain effective control of HIV-1 replication while others do not. Genes implicated in this control include a number associated with the development of classical immune effector responses against HIV-1 infection and others associated with viral entry. CCL3, formerly known as macrophage inflammatory factor 1α (MIP-1α), is a ligand for the HIV-1 co-receptor CCR5 and a chemokine that inhibits HIV-1 replication. The present study was undertaken to assess the effect of *CCL3L* copy number on the control of virus replication and AIDS progression in SIVmac251-infected, Indian-origin rhesus monkeys. We found that *CCL3L* copy number varies in rhesus monkeys. However, the copy number is not predictive of SIVmac251 containment following infection.

## Introduction

Host genetic factors influence susceptibility to HIV-1 replication and progression to AIDS. A number of genes in humans have been shown to impact the development of classical immune effector responses against HIV-1 infection and HIV-1 entry into cells. The genes associated with immune control of HIV-1 include selected MHC class I alleles, including HLA B*5701, HLA B*27 [1], *HLA C* alleles [2], and *HLA Bw4-8OI* in association with *KIR3DS1* [3]. Expression of the delta 32 allelic form of the HIV-1 co-receptor *CCR5* is associated with an inhibition of HIV-1 entry into cells [4]–[6].

CCL3, formerly known as macrophage inflammatory factor 1α (MIP-1α), a ligand for CCR5, is a chemokine with profound HIV-1 inhibitory activity [7]–[9]. The two functional genes coding for CCL3, *CCL3* and *CCL3-like* 1 (*CCL3L1*), map to a narrow region on human chromosome 17. While *CCL3* exists as two copies per diploid genome (pdg), *CCL3L1* arose from *CCL3* through segmental duplication, and the copy number of *CCL3L1* varies among individuals as a consequence of unequal duplications [10], [11]. It has been suggested that more copies of a gene should translate into more protein expression, and higher *CCL3L1* copy number has been correlated with enhanced CCL3 production [11]. Several groups reported that a low *CCL3L1* copy number is associated with an increased risk of acquiring HIV-1 as well as both high viral loads and rapid progression to AIDS following infection [7], [9]. However, a recent series of reports show no effect of *CCL3L1* CNV on HIV-1 infection, viral loads, or progression to AIDS following infection [12]–[14].

CNV at the *CCL3L* loci exists in both chimpanzees and rhesus monkeys [7], [15]. Indeed, a link between *CCL3L* CNV and AIDS susceptibility has recently been reported in rhesus monkeys infected with SIV [15]. The present study was initiated to explore the possibility of an association between *CCL3L* CNV and AIDS in rhesus monkeys.

## Results

**Figure 1:**
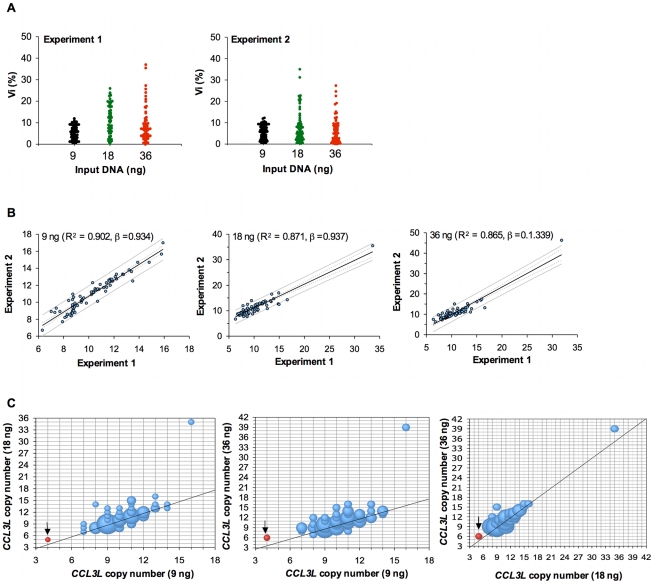
Intra-experiment reproducibility and inter-experiment variability in *CCL3L* copy-number determination. (A) Intra-experiment reproducibility as determined using duplicates for each DNA sample from 84 rhesus monkeys. The results of two separate experiments are shown. (B) Inter-experiment variability. *CCL3L* copy numbers were determined by real-time qPCR using different amounts of input DNA. Correlations between the mean of the unrounded *CCL3L* copy number estimates from two separate experiments are shown. (C) Bubble plots showing the concordance between rounded *CCL3L* copy number estimates determined using different amounts of input DNA. The precision of each assay was determined by the rounded *CCL3L* copy number estimate of from reference sample, the A431 human cell line (black arrow).

This study was done to assess the effect of *CCL3L* CNV on viral load and disease progression following SIVmac251 infection in Indian-origin rhesus monkeys. We first conducted experiments to standardize the technical approach for determining *CCL3L* copy number for this study. We assessed whether *CCL3L* copy number calculated from the *CCL3L*-derived signal in our qPCR assay systematically increased or decreased according to the quantity of input DNA as described by Urban et al. [12]. We evaluated the copy number of *CCL3L* using 18 ng of input DNA by the qPCR method described by Degenhardt et al. [15] and concurrently evaluated *CCL3L* copy number using either 9 ng or 36 ng of input DNA. We explored the reproducibility of this qPCR assay using duplicates for each of 69 DNA samples in each of two separate experiments and evaluating the percent difference (defined as the difference between *CCL3L* copy numbers in the duplicates divided by their average, called Vi) as described by Urban et al. [12]. Higher quantities of input DNA tended to be associated with larger ranges of Vi (Figure 1A); the averages of Vi over all 69 samples and the two experiments were 5.591 (9 ng), 9.681 (18 ng), and 7.725 (36 ng). Assessing the *CCL3L* copy number averaged over the two duplicates, the copy numbers obtained by the two separate experiments had greater Pearson correlations using 9 ng than either 18 ng or 36 ng of input DNA (Figure 1B for related regressions). Thus, the use of more concentrated DNA samples was associated with greater variability between replicates.

We then looked at the average (of 4 numbers – duplicates from the two experiments) of the *CCL3L* copy numbers and regressed the experiments using 18 ng and 36 ng of input DNA on the experiment using 9 ng of input DNA. We found that 18 ng of input DNA underestimated in 14 (20.3%) and overestimated in 20 (29.0%) samples, and also found that 36 ng of input DNA underestimated in 19 (27.5%), and overestimated in 28 (40.6%) samples (Figure 1C). Therefore, we only used determinations of *CCL3L* copy number generated from DNA samples diluted to 9 ng in this study.

Then, to determine the accuracy of our qPCR assay, we assessed the copy number of *CCL3L* in the reference A431 human cell line. The A431 cell line has previously been shown to have two copies of *CCL3L1* and two copies of *CCL3* pdg, for a total copy number of four *CCL3L* [11]. We found that the A431 human cell line had 4 *CCL3L* copies when 9 ng of input DNA was assessed. However, *CCL3L* copy number appeared to be higher than 4 when 18 or 36 ng of input DNA input were assayed (Figure 1C).

Since we have previously shown that Indian-origin and Chinese-origin rhesus monkeys differ substantially in their control of SIVmac251 replication [16], we reasoned that studying genetic determinants of SIVmac251 control in a combined population of both Indian-origin and Chinese-origin rhesus monkeys might introduce a bias into the results. We therefore chose to pursue the present study to evaluate the association of *CCL3L* CNV with the control of SIV replication and AIDS progression in a cohort of SIVmac251-infected Indian-origin rhesus monkeys.

The expression of particular MHC class I alleles in Indian-origin rhesus monkeys is associated with the efficient control of SIV replication and the rate of disease progression following SIV infection [17]–[19]. We also recently demonstrated a central role of *TRIM5* polymorphism in limiting the replication of SIVmac251 in a primate host [20]. Although MHC class I, *TRIM5* and *CCL3L* are located on different chromosomes, it is possible that the alleles of these genes are correlated with each other. If that were the case, a *CCL3L* copy number effect could be confounded or masked by either MHC class I or *TRIM5* alleles associated with SIV control. In order to address this issue, we included the expression of both the MHC class I allele Mamu-A*01 and specific *TRIM5* alleles in our analysis to assess the effect of *CCL3L* CNV on the control of SIVmac251 replication in Indian-origin rhesus monkeys.

**Figure 2:**
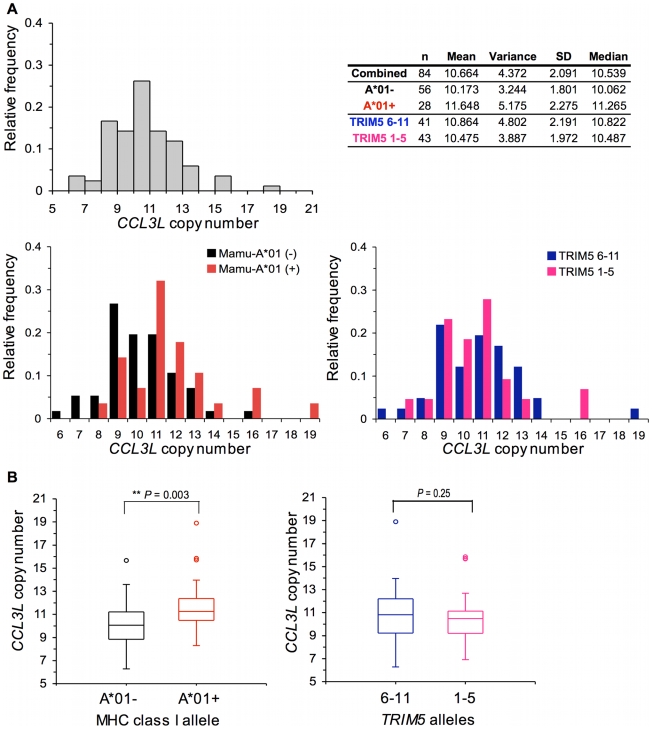
Distribution of *CCL3L* copy number in Indian-origin rhesus monkeys. Copy numbers of *CCL3L* genes were estimated using real-time PCR in 84 Indian-origin rhesus monkeys. (A) Frequency distribution of *CCL3L* copy number. Data are displayed three ways; combining all monkeys, dividing the monkeys into 2 cohorts: one Mamu-A*01− and the other Mamu-A*01+, or dividing the monkeys into 2 cohorts: one of animlas expressing only TRIM5 alleles 1–5 and the other of animals expressing at least 1 TRIM5 allele of the groups 6–11. The mean, variance, standard deviation (SD) and median of the copy number are shown. (B) Boxplots of *CCL3L* copy number in Mamu-A*01− and Mamu-A*01+ animals, and, in one of animlas expressing only TRIM5 alleles 1–5 and the other of animals expressing at least 1 *TRIM5* allele of the groups 6–11. The comparisons were analyzed using the Mann-Whitney U test (two-tailed).

We evaluated *CCL3L* CNV in 84 Indian-origin rhesus monkeys. The distribution of *CCL3L* copy number in this cohort of rhesus monkeys is shown in Figure 2A. We observed extensive variation in *CCL3L* copy number in these animals, with a range of 7 to 19 copies pdg (median 10.539). Data are displayed three ways in Figure 2A: combining all monkeys, dividing the monkeys into 2 cohorts, one Mamu-A*01− and the other Mamu-A*01+, and dividing the monkeys into 2 cohorts, one of animals expressing only *TRIM5* alleles 1–5 and the other of animals expressing at least 1 *TRIM5* allele of the groups 6–11 as previously described [20]. *CCL3L* copy number did not appear to be equally distributed in subgroups defined by Mamu-A*01 and by *TRIM5*. Animals with high *CCL3L* copy number were overpresented among the Mamu-A*01+ monkeys (shift to the right in Figure 2A). Importantly, we found that Mamu-A*01+ rhesus monkeys possessed a significantly greater number of *CCL3L* gene copies than the Mamu-A*01- rhesus monkeys (Mann-Whitney *P* = 0.003). However, the expression of particular *TRIM5* alleles was not significantly associated with the *CCL3L* copy number in the same cohort of rhesus monkeys (Figure 2B).

Using all of these findings to inform our experimental approach, we evaluated associations of *CCL3L* CNV with the *in vivo* control of SIVmac251 replication during the period of acute infection in a cohort of Indian-origin rhesus monkeys following intravenous infection with SIVmac251. Plasma virus RNA levels were assessed on day 14, at the time of peak virus replication, and on day 70, at the time of set-point virus replication following SIVmac251 infection. The peak and set-point plasma SIV RNA levels have been shown to be predictors of disease progression in SIVmac251-infected monkeys [21], [22].

To evaluate the effects of *CCL3L* copy number (used as a continuous variable), Mamu-A*0l, and *TRIM5* alleles (both evaluated as binary variables) separately, we first used single covariate linear regressions of log plasma SIV RNA levels at both peak and set-point. When considered individually, we found that each of the three variables were associated with log peak plasma SIV RNA levels, but Mamu-A*01 was by far the most significant (*P*<0.0001 vs. 0.04 for each of the other two covariates; Table 1). In the regressions for log set-point plasma SIV RNA levels, the expression of specific *TRIM5* alleles was the most significant single covariate (*P* = 0.001) and Mamu-A*01 was significant (*P* = 0.04) but *CCL3L* copy number was not significant (*P* = 0.53).

**Table 1:** Regressions with single covariates.

Trait	Covariate	n	β	R^2^	*P* value
Plasma SIV RNA (Peak)	MHC class I (A*01)	66	−0.646	0.256	**<0.0001**
	*TRIM5*	66	−0.238	0.065	**0.04**
	*CCL3L* copy number	66	−0.052	0.065	**0.04**
Plasma SIV RNA (Set-point)	MHC class I (A*01)	62	−0.760	0.066	**0.04**
	*TRIM5*	62	−0.941	0.183	**0.001**
	*CCL3L* copy number	62	−0.039	0.007	0.53

Estimated coefficient (β) and *P* values are reported for the MHC class I (A*01), *TRIM5* alleles and the *CCL3L* copy number. *R* ***^2^*** values represent the fraction of variation in the plasma SIV RNA levels explained by the single covariate in the model.

When considering several covariates together in the linear regressions, the effect of *CCL3L* copy number was not significantly associated with log peak plasma SIV RNA levels in any model containing Mamu-A*01 (Table 2). *CCL3L* copy number was significant (*P* = 0.02) in the model containing only *TRIM5* alleles, but that model had a much smaller R^2^ than any model involving Mamu-A*01. The effect of *CCL3L* copy number was not significant in any of the models for log set-point plasma SIV RNA levels (with any combination of the other two covariates, Table 2).

**Table 2:** Effect of *CCL3L* copy number in regressions with several covariates.

Trait	Covariate	n	β	*P* value
Plasma SIV RNA (Peak)	MHC class I (A*01)	66	0.0123	0.92
	*TRIM5*	66	−0.058	**0.02**
	MHC class I (A*01) & *TRIM5*	66	−0.007	0.78
Plasma SIV RNA (Set-point)	MHC class I (A*01)	62	0.033	0.48
	*TRIM5*	62	−0.086	0.13
	MHC class I (A*01) & *TRIM5*	62	−0.087	0.54

Estimated coefficient (β) and *P* values are reported for the *CCL3L* copy number after correcting for the expression of MHC class I (A*01) or *TRIM5* alleles or both.

This analysis suggests that *CCL3L* copy number could simply serve as a surrogate for the expression of the MHC class I allele Mamu-A*01 and the early virus control associated with high or low *CCL3L* copy number might be consequence of that MHC class I allele. Therefore, our results show that it is important to control for MHC class I alleles or other factors that might be associated with *CCL3L* copy number in the study.

For power calculations, we considered the residual (Res) log plasma SIV RNA levels when Mamu-A*01 was in the model (i.e., for each animal, the difference between the observed viral load and that predicted by Mamu-A*01). For the peak viral load regressions, with 66 animals there is 80% power to detect as significant the contribution of *CCL3L* to the model if the true correlation between Res and *CCL3L* is at least 0.34. (For comparison, the observed value of the Pearson correlation was 0.01). For the set-point viral load regressions, there is 80% power to detect as significant the contribution of *CCL3L* copy number to the model if the true correlation between Res and *CCL3L* copy number is at least 0.36 (the observed value of the Pearson correlation was 0.13).

**Figure 3:**
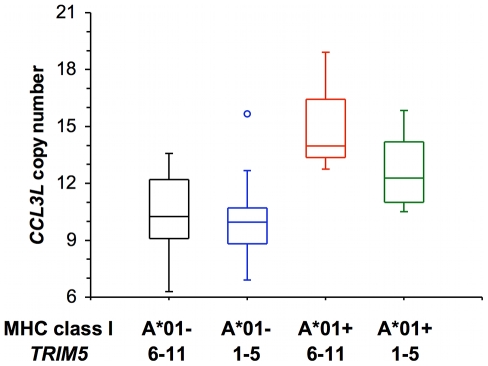
Distribution of *CCL3L* copy number in a cohort of Indian-rhesus monkeys. Rhesus monkeys are divided into 4 groups according to their expression of Mamu-A*01 allele (A*01− and A*01+) and selected *TRIM5* alleles (*TRIM5* 1–5: one expressing only *TRIM5* alleles 1–5, *TRIM5* 6–11: the other expressing only *TRIM5* alleles 6–11).

We also divided the rhesus monkeys used in this study into four separate groups according to the expression of Mamu-A*01 and *TRIM5* alleles by the monkeys as shown in Figure 3. There was a significant difference in *CCL3L* copy number between these 4 groups (Kruskal-Wallis *P* = 0.0017), primarily between Mamu-A*01+ and Mamu-A*01−. The effect of *CCL3L* copy number was not significant (using a linear regression) for either log peak plasma SIV RNA levels or log set-point plasma SIV RNA levels for most of these subgroups. Only for the group of monkeys that expressed Mamu-A*01 and one of *TRIM5* alleles from the group of allele 6–11 was the *CCL3L* copy number coefficient significant, but the number of monkeys in that group was very small (n = 3) (Table 3).

**Table 3:** Comparison of the regression coefficient (β) values and the significance of *CCL3L* copy number from rhesus monkeys expressing various combinations of both Mamu-A*01 and *TRIM5* alleles.

	Groups				
Trait	MHC class I	*TRIM5*	n	β	*P value*
Plasma SIV RNA (Peak)	A*01−	6–11	24	−0.024	0.50
	A*01−	1–5	32	0.025	0.55
	A*01+	6–11	3	−0.121	**0.05**
	A*01+	1–5	7	0.066	0.58
Plasma SIV RNA (Set-point)	A*01−	6–11	23	−0.144	0.09
	A*01−	1–5	29	−0.009	0.96
	A*01+	6–11	3	0.073	0.39
	A*01+	1–5	7	0.166	0.28

β and *P values* are reported for the *CCL3L* copy number.

We assessed whether the quantitation of the copy number of *CCL3L* using a primer/probe set that does not differentiate between *CCL3* and *CCL3L* gene paralogs as described by Degenhardt *et al*. could be subject to systematic biases. To address this possibility, we designed an alternative primer set and probe specific for *CCL3*-*like* gene 1 (*CCL3L1*) using the known rhesus monkey genome sequence (NCBI Reference Sequence: NW_001160084) and found that this primer set and probe are unique for *CCL3L1* by “blastn” analysis (http://www.ncbi.nlm.nih.gov/BLAST/). We again observed extensive variation in *CCL3L1* copy number in a cohort of animals, with a range of 3 to 13 copies pdg (median 8.04) (Supplementary Figure 3 in Text S1). Consistent with the results of CCL3L1 CNV, these results showed that there was a significant difference (Mann-Whitney *P* = 0.04) in the *CCL3L1* copy numbers between Mamu-A*01− and Mamu-A*01+ rhesus monkeys. However, the association between *CCL3L1* copy number and either log peak or log set-point plasma SIV RNA levels was not significant in linear regression analysis (Supplementary Figure 4 in Text S1).

**Figure 4:**
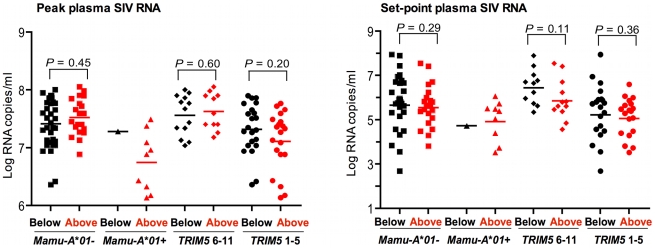
Lack of association of *CCL3L* CNV with virus replication in Indian-origin rhesus monkeys following SIVmac251 infection. The plasma SIV RNA levels were assessed on days 14 and 70 following challenge, representing peak and set-point plasma SIV RNA levels, respectively. The monkeys were divided into 2 groups, one having *CCL3L* copy numbers below the median in this cohort of monkeys (black), and the other having *CCL3L* copy numbers above the median (red). These two groups of rhesus monkeys were subdivided into four separate groups according to their expression of Mamu-A*01 (− or +) and *TRIM5* alleles (6–11 or 1–5). The association of *CCL3L* groups with plasma SIV RNA levels was assessed. The comparisons were analyzed using the Mann-Whitney U test (two-tailed).

We also divided the monkeys into 2 groups according to whether the monkey's *CCL3L* copy number was “below” and “above” the median copy number (10.54) for the entire population of 66 monkeys. To be sure that this comparison was not affected by the expression of Mamu-A*01 or *TRIM5* alleles in the two *CCL3L* groups, we divided each group of monkeys into four separate groups according to their expression of Mamu-A*01 (− or +) and *TRIM5* alleles (6–11 or 1–5) as shown in Figure 4. In this analysis we again found no evidence for an association of *CCL3L* copy number with either peak or set-point plasma SIV RNA levels (Figure 4).

Finally, we assessed whether *CCL3L* copy number groups (Below, < median of 10.54, or Above, ≥ median) are associated with the percent of monkeys dying by day 235 (the follow-up time was chosen ahead of time; after 235 days some monkeys were euthanized and some were used in other experiments) in 45 monkeys from 7 experiments. These percents were compared by Fisher exact test using each covariate (Mamu-A*01, *TRIM5* alleles and *CCL3L*) separately. *CCL3L* groups were not significant (*P* = 0.33); however, Mamu-A*01− status was associated with significantly more frequent death (*P* = 0.04) as was *TRIM5* alleles 6–11 (*P* = 0.005 (Table 4). To be sure that the *CCL3L* comparison was not affected by an imbalance of Mamu-A*01− status or *TRIM5* alleles 6–11 in the two *CCL3L* groups, we also did an exact stratified Fisher test for *CCL3L* groups, stratified separately on Mamu-A*01− status (*P* = 0.99) and on *TRIM5* alleles (*P* = 0.83). There were too few death (especially among Mamu-A*01+ monkeys) to stratify on both Mamu-A*01 and on *TRIM5 alleles* (Table 5).

**Table 4:** Analysis of the survival status on day 235 following SIVmac251 infection.

		Status			
Covariate		Alive	Dead (%)	Estimated RR (95% CI)	*P_F_*
MHC class I	Mamu-A*01−	23	12 (34)	NE	**0.04**
	Mamu-A*01+	10	0 (0)		
*TRIM5*	*TRIM5* 6–11	6	13 (68)	7.2 (1.6–33.8)	**0.005**
	*TRIM5* 1–5	20	6 (23)		
*CCL3L* copy number	Below median (<10.54)	11	6 (35)	2.0 (0.4–9.4)	0.33
	Above median (≥10.54)	22	6 (21)		

The results are reported as number of animals observed.

NE: Not estimable because of zero cells.

**Table 5:** Test of *CCL3L* copy number group stratified on other covariates.

			Status				
Covariate		*CCL3L* copy number group	Alive	Dead (%)	Estimated stratified RR (95% CI)	Fisher Exact stratified test of RR	Test of Homogeneity of RR's
MHC class I	A*01−	Below median (<10.54)	10	6 (38)	1.3 (0.3–6.6)	0.99	1.00
		Above median (≥10.54)	13	6 (32)			
	A*01+	Below median (<10.54)	1	0 (0)			
		Above median (≥10.54)	9	0 (0)			
*TRIM5*	6–11	Below median (<10.54)	2	3 (60)	1.5 (0.3–8.5)	0.83	0.46
		Above median (≥10.54)	4	10 (71)			
	1–5	Below median (<10.54)	8	4(33)			
		Above median (≥10.54)	12	2(14)			

## Discussion

We demonstrated extensive variation in *CCL3L* copy number in a cohort of SIVmac251-infected Indian-origin rhesus monkeys using real-time qPCR. However, we also showed that *CCL3L* copy number co-stratified in this cohort of monkeys with Mamu-A*01, an MHC class I allele associated with SIVmac251 control. Thus, Mamu-A*01+ rhesus monkeys possess a significantly greater number of *CCL3L* gene copies than Mamu-A*01− rhesus monkeys. These findings suggest that *CCL3L* copy number might simply serve as a surrogate for the Mamu-A*01 allele and the association of control of SIV/AIDS pathogenesis associated with high or low *CCL3L* copy number reported by Degenhardt *et al*. might actually be a consequence of the expression of that MHC class I allele.

Degenhardt *et al.* also suggested that *CCL3L* CNV underlies not only a significant inter-individual variation in the rate of progression to AIDS in rhesus monkeys, but also the differences in the pathogenicity of SIVmac251 between Indian-origin and Chinese-origin rhesus monkeys. Indian-origin and Chinese-origin rhesus monkeys certainly differ substantially in their control of SIVmac251 replication [20], [23]. However, these differences in control may be a consequence of other genetic factors. We therefore chose to evaluate the association of *CCL3L* CNV with the control of SIV replication and AIDS progression in a cohort of SIVmac251-infected Indian-origin rhesus monkeys. Exploring a possible association of *CCL3L* CNV with SIVmac251 control in such a defined cohort of rhesus monkeys would eliminate a bias that might be introduced into the results by the diverse genetic factors that may exist in a combined population of rhesus monkey subspecies.

There are a number of potential caveats associated with the findings in the present study. Although more copies of the *CCL3L* gene should translate into a higher level of CCL3L expression, we did not explore whether *CCL3L* copy number influences *CCL3L* mRNA expression in peripheral blood mononuclear cells (PBMCs) isolated from these experimental animals. Moreover, we only evaluated an association of *CCL3L* CNV with the clinical consequences of infection by one SIV isolate (SIVmac251). Since other SIV isolates may make use of a different array of co-receptors to infect cells, it is possible that the consequences of *CCL3L* copy number on SIV control may differ for different SIV isolates [24]. Finally, the interaction between chemokines and their receptors can be promiscuous; a single chemokine can bind multiple receptors and a single receptor can bind to multiple chemokines. CCR5 binds to multiple chemokine ligands as it subserves its biologic functions. These include MIP-1β (*CCL4*) and RANTES (*CCL5*). In humans, there are multiple *CCL4L* genes [25]; whether a similar diversity of these genes exist in rhesus monkeys and contribute to SIV pathogenesis either independently of, or in association with *CCL3L* in monkeys is not known.

*CCL3L* expression level was implicated in the control of HIV-1 replication and AIDS progression in humans as reported by Gonzalez *et al*. [7]. However, results from other recent studies suggest that *CCL3L* copy number is not associated with HIV-1 acquisition, viral loads or progression to AIDS following infection [12]–[14]. The present data are consistent with the latter findings, suggesting that *CCL3L* CNV is not associated with primate immunodeficiency virus containment following infection and, therefore, is not consequential in HIV/SIV pathogenesis.

## Materials and Methods

### Isolation of genomic DNA

Genomic DNA was isolated from freshly isolated PBMCs of 84 Indian-origin rhesus monkeys (66 animals inoculated with SIVmac and 18 uninfected animals) by using the QIAamp DNA kit (Qiagen). This group of monkeys included 56 Mamu-A*01− and 28 Mamu-A*01+ animals. Isolated total DNA quality was verified by average A_260_/A_280_ ratio of 1.84 (range1.77–1.98). These DNA samples were then aliquoted and stored at −20°C until use. We confirmed the integrity of selected stored DNA samples using gel electrophoresis.

### Oligonucleotide primers and probes for real-time PCR

The qPCR primer and probe sequences for *CCL3L* were as described by Degenhardt *et al*. [16]. This primer set does not distinguish between *CCL3* and the *CCL3*-*like* gene paralogs; therefore, *CCL3* and its paralogs are referred to as *CCL3L*.

### Estimation of *CCL3L* copy number using real-time PCR

We determined the gene copy number of rhesus monkey *CCL3L* using the method of Degenhardt *et al*. [15] with a few modifications. Briefly, qPCR was performed using the 7300 Real-Time PCR System (Applied Biosystems) detecting emitted fluorescence as FAM from the probe detecting *CCL3L* and VIC from the probe detecting the *STAT6* gene during amplification. The qPCR included primers and probes with the *Taqman PCR mastermix* (Applied Biosystems). The amount of test DNA sample added to each PCR reaction was between 9–36 ng. Cycling conditions were: initial denaturation at 94°C for 2 min; followed by 40 cycles of 15 sec denaturation at 94°C; and 1 min annealing/extension at 60°C. The *Stat6* gene was used as the internal control. Real-time qPCR results were analyzed using the SDS v1.4.0 software package (Applied Biosystems).

### Generation of standard curves of C_T_ value

Seven serial 1∶2 dilutions (50–0.78 ng) of genomic DNA from A431 cells known to have two copies of *CCL3* and *CCL3L1* pdg were used to generate standard of C_T_ value against log DNA on each PCR plate (96 wells) for *STAT6* present two copies per pdg and *CCL3L*. The values obtained for the target gene *CCL3L* in rhesus monkeys and the normalizer gene, *STAT6*, were similar, which makes *STAT6* gene a good housekeeping standard to estimate of the copy numbers of *CCL3L* in rhesus monkeys. The square of the Pearson correlation coefficient (R^2^) for a standard curve of less than 99% was considered inadequate, and the corresponding PCR plate of DNA samples were repeated. The signal obtained for the test DNA samples always fell on the standard curve range. In Supplementary Figure 1 in Text S1, we reported the calibration curve for the A431 reference sample.

### Absolute quantitation of *CCL3L* copy number based on reference samples

To estimate the absolute *CCL3L* copy number for each sample based on the real-time qPCR results as described by Degenhardt et al., we used the same reference sample, the A431 human cell line. The A431 cell line was shown to have two copies of *CCL3L1* and two copies of *CCL3* pdg, for a total copy number of four *CCL3L* using the qPCR assay. We determined the absolute *CCL3L* copy number in each sample by comparing qPCR results between the experimental and the reference samples. For each qPCR, triplicate wells were set up for *STAT6* and *CCL3L*, C_T_ was determined, and converted into template quantity using standard curves. Copy number is the ratio of the averaged template quantity across the replicates for *CCL3L* to the averaged template quantity of *STAT6*, multiplied by four (the diploid copy number of the A431 cell line including *CCL3L*1 and *CCL3*) or two, respectively. The resulting number was then rounded to the nearest integer value to estimate absolute copy number.

### *In vivo* infection

#### Animals

Indian-origin rhesus monkeys used in the analysis were maintained according to the guidelines of the *NIH Guide to the Care and Use of Laboratory Animals* and the approval of the Institutional Animal Care and Use Committee of Harvard Medical School and the National Institutes of Health. All monkeys (n = 66) used in this retrospective analysis were part of 9 different SIV research experiments. All animals were received 1 ml of a 1∶3000 dilution of this stock by intravenous route. Animals from 2 experiments (n = 21) were excluded from the results of survival status analysis since they were euthanized or used in other experiments soon after day 70 (before any developed AIDS-related illness).

#### Challenge virus

A stock of uncloned SIVmac251 was expanded on human PBMC and titered *in vivo* in rhesus monkeys for use in intravenous challenge studies [26].

### Plasma viral RNA assay

Assays were performed using an ultrasensitive branched DNA amplification assay (Bayer Diagnostics).

### Statistical analysis

All statistical analyses were conducted using the XLSTAT software (Addinsoft) and the StarXact 8 (Cytel) for Fisher exact tests and stratified Fisher exact test, and nQuery Advisor 6.0 (Statistical Solutions) for power calculations for regression. The log_10_ SIV RNA levels in plasma at the time of peak and set-point were compared between groups by use of the exact Mann-Whitney U test as was comparing *CCL3L* copy number between Mamu-A*01 subgroups and between *TRIM5* subgroups. The Kruskal-Wallis test was used to compare *CCL3L* copy number between 4 subgroups (divided by the expression of Mamu-A*01 and *TRIM5* alleles by the monkeys). Linear regressions of the log_10_ plasma SIV RNA at the time of peak and set-point were used separately on each of three covariates (the binary variable Mamu-A*01 and *TRIM5* alleles and the continuous variable *CCL3L* copy number) and also for models of two or three of these variables together. Fisher exact tests were used to compare the percent of monkeys that died by day 235 in various subgroups (defined by *CCL3L* copy number being below or above the median observed and by Mamu-A*01 and *TRIM5* alleles). Exact estimated relative risk (RR, similar to odds ratio but odds ratio can only be estimated when the true prevalence is known) and 95% confidence intervals (CIs) for RR were provided. Exact tests of association of CCL3L copy number groups and the probability of dying by day 235 stratified by Mamu-A*01 and *TRIM5* alleles and the related stratified RR and test of homogeneity of RR used the method of Zelen [27]. P values were two-tailed and considered significant when <0.05, no corrections for multiple comparisons were used.

## Supporting Information

Text S1 Supplemental methods and figures.
(0.82 MB PDF)
