# Supplementary material for: Contributions of Mamu-A*01 Status and TRIM5 Allele Expression, But Not CCL3L Copy Number Variation, to the Control of SIVmac251 Replication in Indian-Origin Rhesus Monkeys
Source: PLoS Genet. 2010 Jun 24;6(6):e1000997. doi: 10.1371/journal.pgen.1000997 (PMC2891712; doi:10.1371/journal.pgen.1000997)

Supplemental Methods

CCL3L1 specific primers and probes for real-time PCR

The primer set and probe specific for *CCL3L1* were designed using the known rhesus monkey genome sequence (NCBI Reference Sequence: NW_001160084) and were found to be unique for *CCL3L1* by “blastn” analysis (http://www.ncbi.nlm.nih.gov/BLAST/).

The oligonucelotide sequences used for *CCL3L1* were:

Forward: 5′-CAGGCTCATTCTCTTCCTTTCTCT-3′,

Reverse: 5’-GCTGCTGGTCTCAAAGTAGTCA-3′,

Probe: 5′-FAM- TGCTGACACGCCGACCTCCTGCT-BHQ-3′.

As these oligonucleotide sequences are based on the rhesus monkey reference sequence (Mmul_051212), we tested the specificity of the primers by PCR using genomic DNA obtained from the A431 cell line.

Preparation of *CCL3L*, *CCL3L1* and *STAT6* standards

For absolute quantitation, plasmid DNA standards were created for each gene by using primer sets encompassing the sequence to be amplified in the real-time PCR reaction. Oligonucleotide primers for rhesus monkey *CCL3L*, *CCL3L1* and *STAT6* were:

CCL3F: 5’-GGGGCAGGTGTTACAGAGTCAGGA-3’,

CCL3R: 5’-TCAGGCACTCAGCTCCAGGTC-3’,

CCL3L1F: 5’- GAAGAGTCAAGGGGAAAGAAGGAA-3’,

CCL3L1R: 5’-CTGGCTGCTGGTCTCAAAGTAGTC-3’,

STAT6F: 5’- CAGGAAGAAGGGGTGGCATCAACT-3’,

STAT6R: 5’- GGGGCCTAGGGAAAGAAAACAGAC-3’

PCR was used to amplify fragments from genomic DNA isolated from the rhesus monkey fibroblast cell line (FRhK-4). Reaction conditions were 30 cycles of 15 sec denaturation at 94°C and 30 sec annealing at 55°C, and 1 min extension at 68°C. The single amplified PCR product was verified based on size in a 1% agarose gel under UV light. The amplified products were recovered and purified using a PCR purification kit (Qiagen). The amplicons were ligated directly into the TOPO Blunt vector (Invitrogen) generating pCCL3L, pCCL3L1, and pSTAT6, respectively. Clones containing inserts were identified by restriction enzyme analysis and the expected sequences were verified by sequencing. After plasmid maxi preparation (Qiagen), the concentration of plasmid dsDNA was measured with a spectrophotometer.

Generation of standard curves of C_T value

A431 cell line standard:

Seven serial 1:2 dilutions (50 – 0.78 ng) of genomic DNA from A431 cells known to have two copies of *CCL3* and *CCL3L1* pdg were used to generate standard of C_T value against log DNA on each PCR plate (96 wells) for *STAT6* present two copies per pdg and *CCL3L*. The values obtained for the target gene *CCL3L* in rhesus monkeys and the normalizer gene, *STAT6*, were similar, which makes *STAT6* gene a good standard to estimate of the copy numbers of *CCL3L* in rhesus monkeys. The square of the Pearson correlation coefficient (R^2) for a standard curve of less than 99% was considered inadequate, and the corresponding PCR plate of DNA samples were repeated. The signal obtained for the test DNA samples always fell on the standard curve range. In Figure S1 in Text S1, we report the calibration curve for the A431 reference sample. Ct values for

standard values of *CCL3L* and *STAT6* from genomic DNA from A431 cells ranging from 0.78 to 50 ng fell along straight semi-log trendlines with R^2 value of 0.9993 and 0.9992, respectively (Fig. S1A, B in Text S1). The gradient of the standards plot for *CCL3L* and *STAT6* was -3.5043 and -3.5237, respectively. From these gradients, the PCR efficiency of *CCL3L* and *STAT6* was calculated to be 92.93% and 92.22%, respectively considering that the theoretical efficiency equation: Efficiency (%) = $(10^{-1/\text{gradient}} - 1) \times 100$.

Plasmid DNA standard:

The number of copies per unit volume was calculated using the molecular weight of each plasmid dsDNA and Avogadro's constant. The stock solutions of each plasmid dsDNA were serially diluted to generate standard of C_T value against log [copies] ($1\text{-}10^8$) with each step differing by 10 fold.

Quality control assessment

We analyzed the results from the real-time qPCR for precision, reproducibility and consistency using a different standard. To confirm that the real-time qPCR absolute copy numbers estimated using the human cell line A431 as a reference sample were accurate, we estimated *CCL3L/CCL3L1* copy numbers using plasmid dsDNA standards. We first assessed the inter-experiment variability by comparing the estimates of *CCL3L* copy numbers from two separate experiments performed on different days. Linear regression of experiment 1 on experiment 2 demonstrated a high degree of correlation between estimates and a slope very close to unity calculated from the plasmid DNA standard ($R^2 = 0.934$, $\beta = 0.959$) (Fig. S2A in Text S1). Fig. S2B in Text S1 shows strong correlation between mean copy number estimates from two separate experiments

determined using different standards (R^2 = 0.827, β = 0.982), confirming the absolute *CCL3L* copy number estimates assayed using real-time qPCR.

Estimation of *CCL3L1* Copy number using Real-Time PCR

We then evaluated *CCL3L1* copy number in the same cohort of rhesus monkeys. We observed extensive variation in *CCL3L1* copy number in these animals, with a range of 3 to 13 copies pdg (Fig. S3 in Text S1). Consistent with the results of *CCL3L* CNV, these results showed that there was a significant difference in the distributions of the *CCL3L1* copy number between *Mamu-A*01*- and *Mamu-A*01*+ rhesus monkeys. *Mamu-A*01*+ rhesus monkeys had a significantly greater *CCL3L1* copy number than *Mamu-A*01*- animals (Fig. S3 in Text S1).

We also assessed whether there are associations between *CCL3L1* copy number and either peak or set-point plasma virus RNA levels during the period of acute SIVmac251 infection. Consistent with the previously demonstrated absence of an effect of *CCL3L* copy number on plasma SIV RNA levels, we found no evidence for an association between *CCL3L1* copy number and either peak or set-point plasma SIV RNA levels in these monkeys (Fig. S4 in Text S1).

Supplemental Figures

Figure S1. Standard curves and amplification plots for *CCL3L and STAT6*

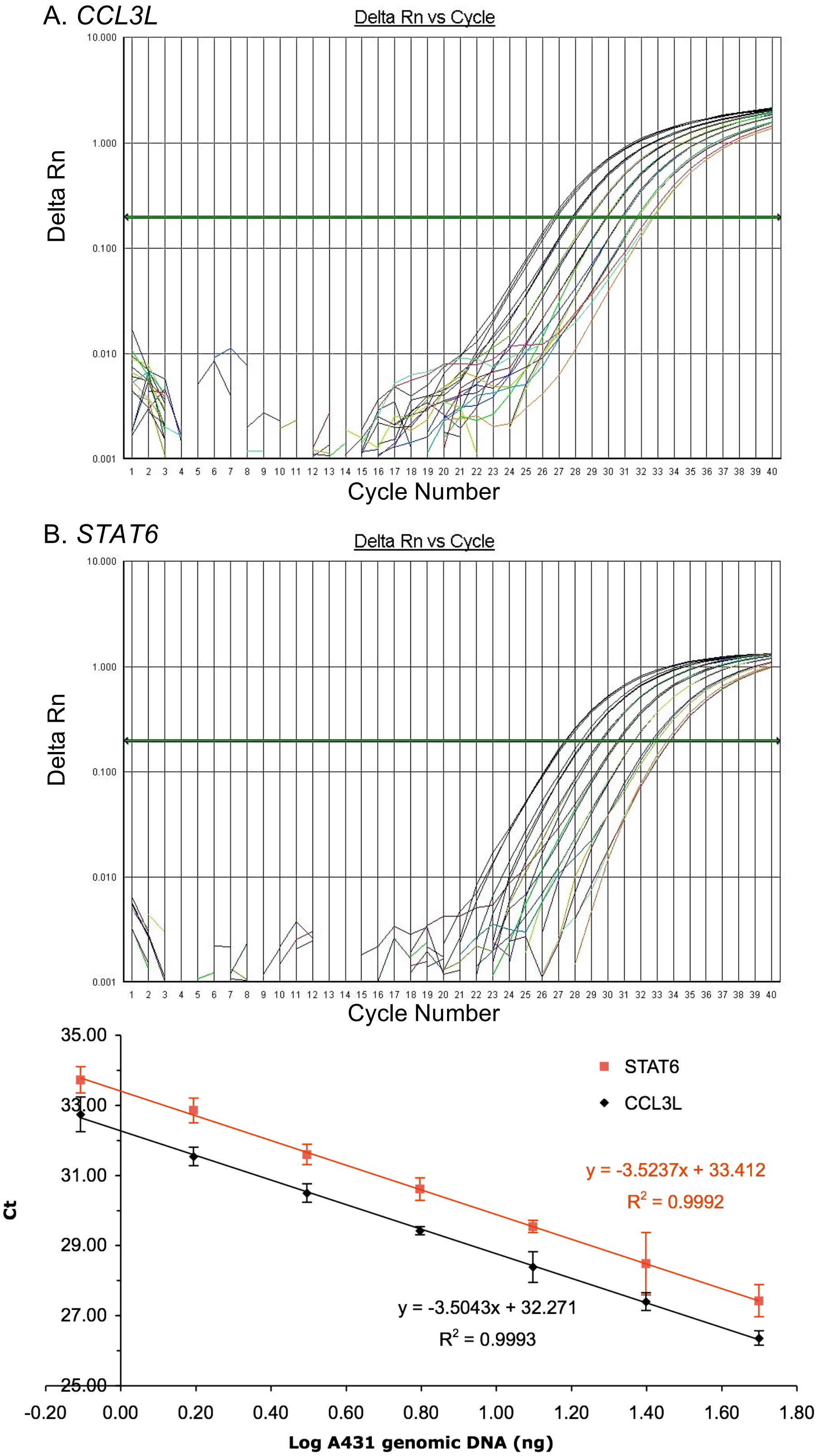

Figure S2. Quality control of real-time qPCR assay used to estimate *CCL3L* copy numbers

A. Plasmid DNA standard

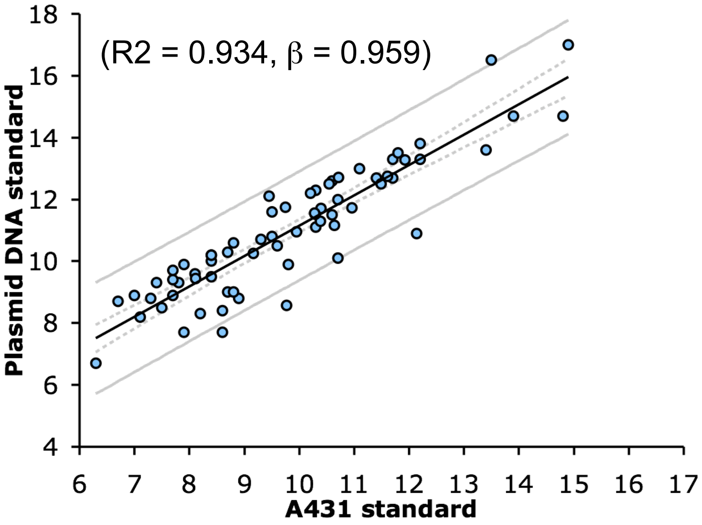

B. Concordance of *CCL3L* copy number estimates

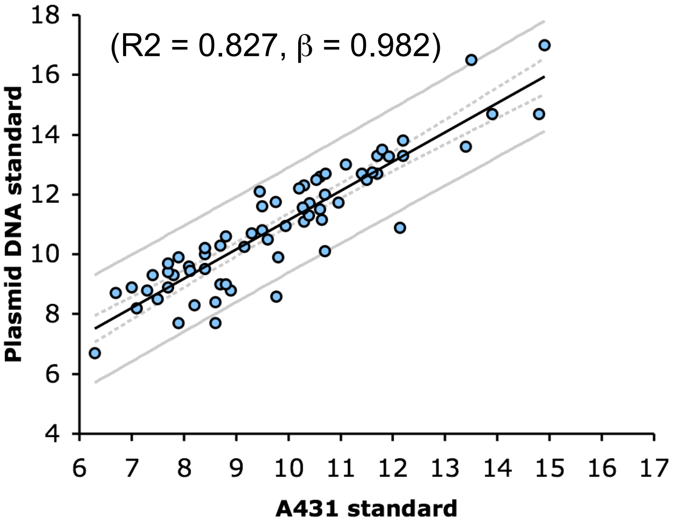

Figure S3. *CCL3L1* copy number variation in Indian-origin rhesus monkeys. Copy numbers of *CCL3L1* genes were estimated using real-time qPCR in 84 Indian-origin rhesus monkeys. A. Frequency distribution of *CCL3L1* copy number in this cohort of monkeys. The mean, variance, standard deviation (SD) and median of the copy numbers are shown. B. Boxplots of *CCL3L1* copy numbers in *Mamu-A*01*- and *Mamu-A*01+* animals. The comparisons were analyzed using the Mann-Whitney U test (two-tailed).

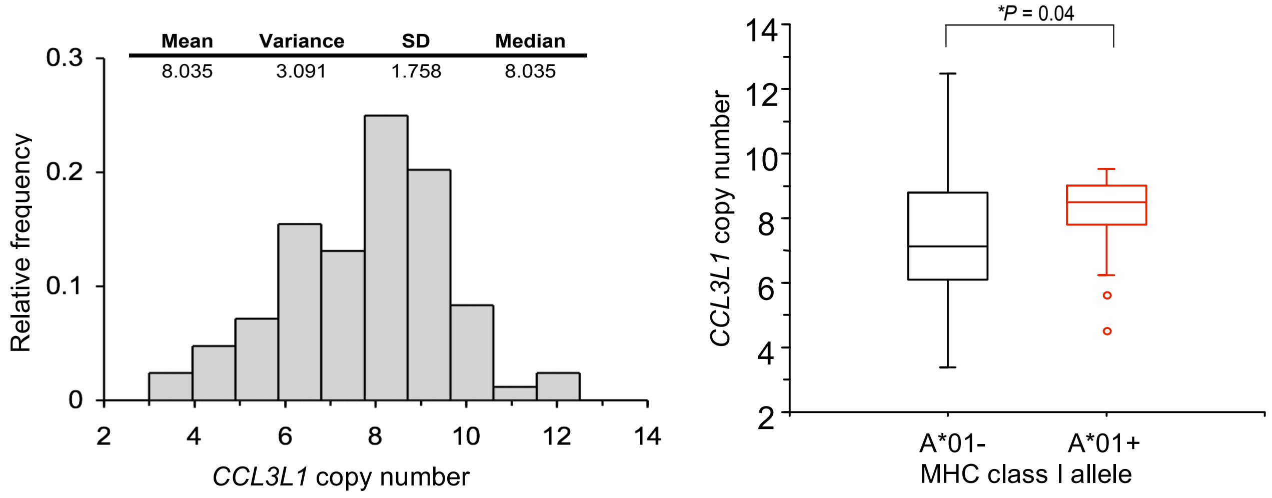

Figure S4. Lack of association of *CCL3L* copy number variation with virus replication following SIVmac251 infection. Linear regressions of peak (day 14) and set-point (day 70) plasma SIV RNA levels following infection with *CCL3L1* copy numbers were analyzed.

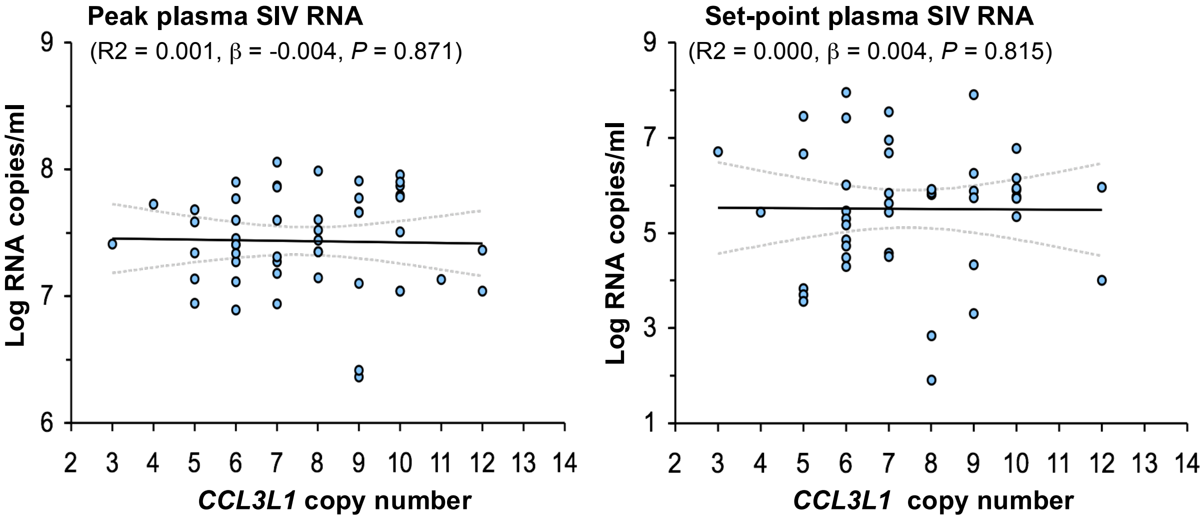

Supplement: Text S1 — Supplemental methods and figures. (0.82 MB PDF) [file pgen.1000997.s001.pdf]
